# Pharmacokinetic and pharmacodynamic similarity between SAR341402 insulin aspart and Japan-approved NovoRapid in healthy Japanese subjects

**DOI:** 10.1038/s41598-021-02410-z

**Published:** 2021-11-25

**Authors:** Masanari Shiramoto, Tatsuya Yoshihara, Wolfgang Schmider, Hiroki Takagi, Irene Nowotny, Miyuki Kajiwara, Hideya Muto

**Affiliations:** 1 SOUSEIKAI Hakata Clinic, Fukuoka, Japan; 2 Sanofi-Aventis Deutschland GmbH, Frankfurt, Germany; 3 Sanofi K.K, Tokyo, Japan; 4 Present Address: Kashiihara Hospital, Fukuoka, Japan; 5 Present Address: Novartis Japan, Tokyo, Japan

**Keywords:** Drug development, Hormonal therapies, Type 1 diabetes, Type 2 diabetes

## Abstract

This study compared the pharmacokinetic and glucodynamic profiles of biosimilar SAR341402 insulin aspart to Japan-approved insulin aspart (NovoRapid) in healthy Japanese males. In this single-center, randomized, double-blind, single-dose, two-period, crossover study, subjects received 0.3 U/kg of SAR341402 or NovoRapid before undergoing a 10 h euglycemic clamp procedure. Plasma insulin aspart concentrations and blood glucose levels were measured, and glucose infusion rates (GIRs) were assessed. Primary endpoints were maximum plasma insulin aspart concentration (INS-C_max_), area under the plasma insulin concentration–time curve to the last quantifiable concentration (INS-AUC_last_), area under the GIR–time curve during the clamp (GIR-AUC_0–10 h_), and maximum GIR (GIR_max_). Forty subjects were randomized with 39 completing both treatment periods. Pharmacokinetic exposure showed a mean ratio between products of 1.00 (90% confidence interval [CI] 0.94–1.05) for INS-C_max_ and 1.02 (90% CI 1.00–1.04) for INS-AUC_last_. Glucodynamic activity showed a mean ratio between products of 1.00 (95% CI 0.93–1.06) for GIR-AUC_0–10 h_ and 1.01 (95% CI 0.95–1.08) for GIR_max_. The 90% CIs for pairwise treatment ratios were within the predefined equivalence range of 0.80–1.25. Both treatments were well tolerated. We concluded that similar pharmacokinetic exposure and glucodynamic potency were shown for SAR341402 and NovoRapid in healthy Japanese males.

## Introduction

SAR341402 (SAR-Asp; Sanofi, Paris, France) is a rapid-acting insulin aspart product^1^, developed in accordance with relevant United States (US), European (EU), and Japanese guidelines for development of biosimilar insulin products^2–6^. Insulin aspart is the active ingredient of an insulin analog product available in Japan and EU as NovoRapid, and as NovoLog in the US. SAR-Asp has an amino acid sequence and structure identical to NovoLog/NovoRapid (NN-Asp; Novo Nordisk, Bagsværd, Denmark) and is formulated at a concentration of 100 U/mL. NN-Asp has been approved for use in adults, adolescents, and children with diabetes in many countries, including Japan, for ~ 20 years^7,8^.

Similar pharmacokinetic exposure and pharmacodynamic activity were shown for SAR-Asp versus both US-reference and EU-reference NN-Asp in a euglycemic clamp study in subjects with type 1 diabetes (T1D)^9^. Subsequently, similar efficacy, safety and immunogenicity of SAR-Asp and NN-Asp were reported in a multinational, randomized phase 3 study among patients with T1D and type 2 diabetes (T2D) using insulin glargine 100 U/mL (Lantus) as the basal insulin^10,11^. SAR-Asp and NN-Asp were also well-tolerated in insulin pump users with T1D treated for 4-weeks^12^.

For approval of any biosimilar insulin in Japan, local guidelines^6^ state that it is necessary to show comparability in pharmacokinetic exposure and pharmacodynamic activity versus the locally available reference product. The present study was designed to show similar pharmacokinetic and glucodynamic profiles for SAR-Asp versus Japan-reference NovoRapid (hereafter called NN-Asp-Jp) in healthy Japanese subjects.

## Subject and method

### Subjects

Subjects were enrolled at Hakata Clinic and included Japanese males aged 20–55 years (both inclusive) with a body mass index of 18.0–28.0 kg/m^2^, certified as healthy by a comprehensive clinical assessment that included his past medical history, normal vital signs, normal 12-lead electrocardiogram (ECG) parameters and laboratory parameters within the normal ranges.

### Study design

This was a randomized, single-center, double-blind, single-dose, 2-treatment, 2-period, crossover study undertaken in 2018–2019 (trial registration: www.clinicaltrials.jp, identifier: JapicCTI-205380, registered 20/07/2020). Subjects received each treatment once. The trial was approved by an ethical review board (Hakata Clinic, Fukuoka, Japan) and conducted in accordance with the principles of the Declaration of Helsinki and the International Conference on Harmonization guidelines for Good Clinical Practice. Written informed consent was provided by all subjects before study entry.

Following a screening visit 3–28 days before the first period, subjects enrolled into the study were admitted to the study clinic on the day before dosing. On the day of treatment, they were randomized (computer-generated by sponsor) to one of the two treatment sequences (see Supplementary Fig. S1). Subjects received a single subcutaneous dose of 0.3 U/kg SAR-Asp or NN-Asp-Jp in the first treatment period in randomized order followed by the other drug in the second treatment period. A wash-out period of 7–18 days separated each treatment period, with an end-of-study visit performed 4–8 days after the last dose.

### Treatments

SAR-Asp (100 U/mL) solution for injection was manufactured by Sanofi (Frankfurt, Germany), with NN-Asp-Jp provided as the commercially available formulation. To maintain double-blinding (with respect of the Investigator, subject and Sponsor) and consistency of dosing methodology, an independent pharmacist at the study site was responsible, with an assistant, for preparing the treatments for each subject.

### Bioanalytical methods

Venous blood samples for pharmacokinetic analysis were collected before dosing of study drug, and then every 15 to 60 min during the 10 h clamp period. Samples were centrifuged within 20 min of collection. Plasma was collected and kept frozen (− 60 to − 80 °C) until analysis. Plasma concentrations of SAR-Asp and NN-Asp-Jp were analyzed by using a validated liquid chromatography-tandem mass spectrometry (LC–MS/MS) assay at Syneos Health (Quebec, Canada). Plasma concentrations within the validated concentration range (100–8000 pg/mL) were used to calculated pharmacokinetic parameters. Inter-assay precision (% coefficient of variation [CV]) and inter-assay accuracy (%bias) during validation were ≤ 15%.

### Pharmacodynamic evaluation using euglycemic clamp

Each dosing visit included a 10 h automated euglycemic clamp procedure (STG-55, Artificial Endocrine Pancreas; Nikkiso Co, Ltd, Tokyo, Japan)^13^. The premise of performing the clamp procedure is that the blood glucose lowering effect of an administered insulin is antagonized by a variable infusion of glucose so that blood glucose is maintained (or clamped) at a target level^2,14^. The metabolic profile of the investigated insulin is characterized by the glucose infusion rate (GIR) needed to keep blood glucose as close as possible at its predefined target level during the glucose clamp^15^.

Following an overnight fast of at least 10 h, subjects were connected to the clamp device and their baseline blood glucose was determined (via glucose oxidase sensors that measure whole-blood glucose levels) before dosing of the study drug. For each subject, this was calculated as the mean of four glucose measurements at 30, 20, 10 and 1 min before administration of the study drugs. The clamp procedure was not performed in those subjects having a baseline glucose level less than 70 mg/dL (3.92 mmol/L). After dosing, onset of insulin action in each subject was when the blood glucose dropped below the target level of the clamp procedure, which was when it was 5 mg/dL (0.28 mmol/L) less than their fasting baseline value^2^.

Blood glucose levels were measured in 1-min intervals, and using a predefined algorithm the clamp device automatically administered a variable infusion of 10% glucose in response to changes in glucose to maintain each subject at their target glucose level. The GIR, being the amount of external glucose needed to keep a subject’s blood glucose concentration at its target level, was continuously measured, and recorded by the STG-55 device. GIR profiles reflected the metabolic effects of SAR-Asp and NN-Asp-Jp over time.

### Safety assessment

The safety and tolerability of single doses of SAR-Asp and NN-Asp-Jp were assessed by 12-lead ECG, vital signs, routine laboratory parameters, physical examination, and reporting of adverse events (AEs). AEs were classified using MedDRA (Medical Dictionary for Regulatory Activities) 21.1. The safety population included all randomized patients who received at least one dose of study insulin, analyzed according to the treatment received. The treatment-emergent AE period included the time from the first dose of study drug up to 72 h after the last dose in each treatment period.

### Pharmacokinetic and pharmacodynamic parameters

The pharmacokinetic analysis dataset included subjects who completed at least one treatment period, had measurable insulin aspart concentrations and no major or critical deviations. Parameter estimates for SAR-Asp and NN-Asp-Jp were calculated by using standard noncompartmental methods with Phoenix WinNonlin 8.1 (Certara, Princeton, NJ). Area under the plasma insulin aspart concentration–time curve was calculated by the trapezoidal method from 0 to the time of the last concentration above the limit of quantification (INS-AUC_last_) and extrapolated to infinity (INS-AUC_inf_). Maximum plasma insulin aspart concentration observed (INS-C_max_) and INS-AUC_last_ were the primary pharmacokinetic endpoints of the study. INS-AUC_inf_ was a secondary endpoint.

The GIR over time was the primary pharmacodynamic parameter measured during the clamp procedure. The area under the body weight-standardized GIR time curve [GIR-AUC] measured insulin mediated glucose uptake into tissues. Subjects completing at least one clamp procedure with no major or critical deviations were included in the pharmacodynamic analysis dataset. Individual GIR values in each treatment group were standardized for body weight and subjected to a smoothing procedure using a locally weighted scatterplot smoothing (LOESS) function (SAS, PROC LOESS, factor 0.06). A smoothing factor of 6% was used based on the expected morphology of the GIR-profiles. Individual blood glucose levels were also subjected to a smoothing procedure. Fitted data were used to calculate the pharmacodynamic parameters, including the primary parameters of maximum smoothed body weight standardized GIR (GIR_max_) and GIR-AUC over 10 h (GIR-AUC_0–10 h_), and the secondary parameter of time to reach GIR_max_ (GIR-t_max_).

Parameters used to assess clamp quality included the mean and individual %CV of blood glucose measurements during euglycemia, and the absolute difference of individual mean blood glucose measurements from the clamp target level, as described previously^9^.

### Statistical analyses

The study aimed to show similarity (equivalence) in pharmacokinetic exposure and glucodynamic activity of a single dose (0.3 U/kg) of SAR-Asp to NN-Asp-Jp under fasting conditions. To achieve this, 26 and 32 evaluable subjects, respectively, were required. These calculations were based on estimates of within-subject variability from a prior SAR-Asp study in subjects with T1D^9^, and those reported in applicable studies performed in healthy subjects^16,17^. A within-subject standard deviation (SD) of 0.175 and 0.180 was assumed for the natural log-transformed pharmacokinetic (INS-C_max_ and INS-AUC_last_) and pharmacodynamic (GIR_max_ and GIR-AUC_0–10 h_) parameters, respectively, for a true treatment ratio between the two formulations of 0.93 and 1.07, respectively. The planned sample sizes provided at least 90% power to show equivalence with a type 1 error of 5% and 2.5% for the pharmacokinetic and pharmacodynamic parameters, respectively. To allow for dropouts, the study planned to recruit at least 36 individuals (18 per sequence).

Log-transformed pharmacokinetic and pharmacodynamic parameter estimates for SAR-Asp and NN-Asp-Jp were analyzed using a linear mixed-effects model with period, sequence, and treatment as fixed effects and subject as a random effect. For each parameter, the model-based difference in treatment means along with the confidence limits (90% for pharmacokinetic parameters, 95% for pharmacodynamic parameters) was back-transformed to provide estimates for the ratio of geometric means (gMean) between treatments (SAR-Asp/NN-Asp-Jp) and the corresponding confidence limits. Similarity for the pharmacokinetic parameters (INS-AUC_inf_ and INS-C_max_) and bioequipotency for the pharmacodynamic parameters (GIR-AUC_0–10 h_ and GIR_max_) was concluded if the confidence limits (90% and 95% confidence intervals [CIs], respectively) of the treatment ratios were entirely within the 0.80–1.25 equivalence interval, in agreement with regulatory guidance^2,18^. Data was analyzed using SAS v9.4 (SAS Institute, Cary, NC).

## Results

Forty Japanese male subjects were randomized and treated, with thirty-nine completing both treatment periods. One subject withdrew from the study because of an AE (road traffic accident) during the washout period before dosing in Period 2. The treatment sequence of this subject was NN-Asp-Jp/SAR-Asp and therefore the pharmacokinetic and pharmacodynamic populations for SAR-Asp were missing. Clamp data during period 2 from five subjects were excluded because of operational errors of the devices (two during SAR-Asp treatment and three during NN-Asp-Jp treatment). In view of these operational errors, an additional four subjects were included in the study. Pharmacodynamic parameters for SAR-Asp and NN-Asp-Jp were available for 36 and 38 subjects, respectively. Baseline characteristics of the included subjects are given in Table 1.

**Table 1:** Baseline characteristics of the study population (safety population).

Characteristics	All subjects (n = 40)
Age (years)	24.5 ± 6.0 [20–45]
Male, *n* (%)	40 (100%)
Race, Japanese, *n* (%)	40 (100%)
Body weight, kg	62.35 ± 6.28 [52.4–78.3]
Body mass index, kg/m^2^	20.87 ± 1.74 [18.1–25.0]

All average data are mean ± SD [min–max].

### Pharmacokinetics

**Figure 1:**
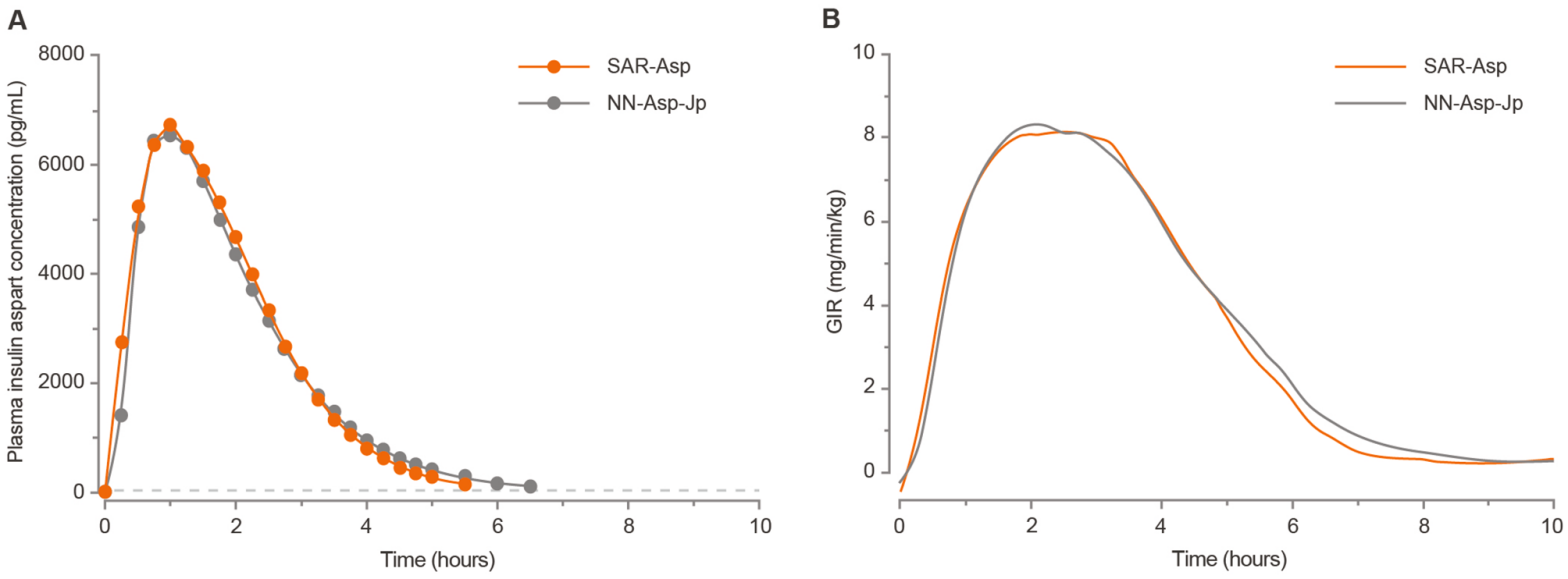
Linear arithmetic mean insulin aspart plasma time profiles (**A**), mean smoothed LOESS fits of body weight standardized GIR versus time profiles (GIR values less than 0 at baseline are results of the LOESS smoothing) (**B**). The horizontal line in (**A**) is the lower level of quantification (100 pg/mL). *GIR* glucose infusion rate, *LOESS* locally weighted scatterplot smoothing.

Single 0.3 U/kg doses of SAR-Asp and NN-Asp-Jp resulted in mean insulin concentration-versus-time profiles that were virtually superimposable (Fig. 1A). The extent of exposure, indicated by gMean INS-AUC_last_ and INS-C_max_ values, was similar between the groups (Table 2), with the gMean ratios close to 1 and the corresponding 90% CIs for each parameter contained within the predefined equivalence interval (0.80–1.25). This confirmed equivalent exposure of the two treatments. The secondary endpoint AUC_inf_ showed similar results to AUC_last_. Low to moderate between-subject variability in the pharmacokinetic parameters was observed, with CVs between 26 and 32%.

**Table 2:** Primary and secondary pharmacokinetic and pharmacodynamic endpoints.

Endpoint	SAR-Asp		NN-Asp-Jp		Mean ratio SAR-Asp/NN-Asp-Jp Point estimates (90% CI PK and 95% CI PD)^a^
	N	gMean (% CV)	N	gMean (% CV)	
**PK endpoints**					
INS-C_max_, pg/mL	39	6690 (32)	40	6690 (31)	1.00 (0.94 to 1.05)
INS-AUC_last_, pg·h/mL	39	15,300 (27)	40	15,100 (27)	1.02 (1.00 to 1.04)
INS-AUC_inf_, pg·h/mL	39	15,400 (26)	40	15,200 (27)	1.02 (1.00 to 1.03)
**PD endpoints**					
GIR-AUC_0-10 h_, mg/kg	36^b^	2054.12 (22.2)	38^c^	2098.06 (23.5)	1.00 (0.93 to 1.06)
GIR_max_, mg/(kg·min)^d^	36^b^	8.70 (20.9)	38^c^	8.72 (21.0)	1.01 (0.95 to 1.08)

*% CV* percent coefficient of variation, *CI* confidence interval, *GIR* body weight standardized glucose infusion rate, *GIR-AUC*_*0–10 h*_ area under the body weight-standardized GIR rate vs time curve from 0 to 10 h, *GIR*_*max*_ maximum smoothed body weight standardized GIR *gMean* geometric mean, *INS-AUC*_*last*_ area under the drug plasma concentration–time curve from time 0 to the time of the last quantifiable data point, *INS-AUC*_*inf*_ area under the drug plasma concentration–time curve from time 0 to infinity, *INS-C*_*max*_ maximum insulin aspart concentration in plasma, *PD* pharmacodynamic, *PK* pharmacokinetic.

^a^90 and 95% CI for the pairwise treatment ratios.

^b^Clamp data from 3 subjects in Period 2 were excluded due to operational errors with the device.

^c^Clamp data from 3 subjects in Period 2 were excluded due to operational errors with the device.

^d^GIR_max_ determined from smoothed GIR data (LOESS method, tension 0.06).

### Pharmacodynamics

In the pharmacodynamic analysis, the mean smoothed bodyweight normalized GIR profiles during the euglycemic clamp procedure (from time 0 to 10 h postdose) were nearly superimposable for SAR-Asp and NN-Asp-Jp (Fig. 1B). The overall pharmacodynamic effects of both treatments were similar, displaying a short time-action profile.

The extent of glucose-lowering, indicated by gMean GIR-AUC_0–10 h_ and GIR_max_ values, was similar for SAR-Asp and NN-Asp-Jp (Table 2), with the gMean ratios close to 1 and the corresponding 95% CIs for each parameter entirely within the equivalence interval (0.80–1.25). This confirmed equipotency of the two treatments. Between-subject variability for GIR-AUC_0–10 h_ and GIR_max_ was low, as shown by CVs between 21 and 24%. The median GIR-t_max_ for SAR-Asp (2.82 h) was similar to that for NN-Asp-Jp (2.35 h).

### Performance of the clamp

Several parameters were used to assess clamp quality during euglycemia (Table 3). Individual mean blood glucose levels were similar for both treatments (mean values of 79.51 and 79.89 mg/dL for SAR-Asp and NN-Asp-Jp, respectively). Individual variability of blood glucose measurements was low with median CV% values of 7.05% and 5.90% for SAR-Asp and NN-Asp-Jp, respectively. Similarly, absolute differences between individual mean blood glucose measurements and the blood glucose target level were low (mean of 2.31 mg/dL for SAR-Asp and 1.74 mg/dL for NN-Asp-Jp).

**Table 3:** Performance of clamp during euglycemia. *BG* blood glucose, *CV* coefficient of variation, *SD* standard deviation.

Parameter and unit	SAR-Asp (n = 36)	NN-Asp-Jp (n = 38)
**Individual mean of BG concentration (during euglycemia) (mg/dL)**^**a**^		
Mean ± SD	79.51 ± 6.12	79.89 ± 6.50
Median (range)	81.40 (68.1–93.0)	80.30 (68.4–97.8)
**Individual CV% of BG (during euglycemia) (%)**^**a**^		
Mean ± SD	8.93 ± 5.52	8.49 ± 5.28
Median (range)	7.05 (3.1–23.6)	5.90 (2.4–21.6)
**Absolute deviation of individual mean BG from clamp level (during euglycemia) (mg/dL)**^**a**^		
Mean ± SD	2.31 ± 1.23	1.74 ± 1.49
Median (range)	2.15 (0.2–5.7)	1.35 (0.1–6.1)

^a^Euglycemia starts with dosing and ends with the last value of the smoothed BG concentration curve at or below the predetermined target blood glucose concentration for each individual subject, as described in the methods. Clamp level (BG target) for each subject was 5 mg/dL (0.28 mmol/L) below the subject’s baseline concentration.

### Safety and tolerability

Single doses of both insulin aspart products were well-tolerated. There were no serious AEs, AEs of special interest or treatment-emergent AEs reported during the study. One subject (sequence of NN-Asp-Jp/SAR-Asp) reported AEs of back pain and neck pain following a road traffic accident during the washout (post treatment) period following Period 1 and withdrew his consent for further participation before dosing in Period 2. A few predefined potentially clinically significant abnormalities in laboratory tests and ECG parameters were reported but with no noticeable differences between SAR-Asp and NN-Asp-Jp. The majority of the abnormalities in laboratory values and ECG parameters were present at baseline prior to administration of SAR-Asp and NN-Asp-Jp. The investigator assessed that none of abnormalities were clinically significant.

## Discussion

In this crossover study in healthy Japanese males, single doses of SAR-Asp insulin aspart solution showed similar pharmacokinetic exposure and glucodynamic activity to the commercially available NN-Asp-Jp formulation, using a euglycemia clamp device to measure insulin action. Between-subject variability estimates for both treatments were low, and both were well tolerated.

The design of this crossover clamp study, involving administration of single-doses of each treatment, is consistent with regulatory guidance for the evaluation of biosimilar insulins^2^. A similar design was used in a trial that compared insulin exposure and pharmacodynamic activity for SAR-Asp with US-reference NN-Asp and EU-reference NN-Asp^9^. We assessed insulin exposure from the plasma insulin concentration–time profiles and insulin activity as glucose utilization during the euglycemic clamp. The crossover design allowed each subject to receive both treatments, so that a comparison between the two treatments could be made on the same subject. This allowed within-subject comparisons of pharmacokinetic and glucodynamic parameters between the two products to minimize potential within- and between-subject variability.

The study was conducted in normal-weight healthy subjects as this provides a homogeneous population that is sensitive to insulin, thereby enabling detection of any potential treatment-related differences^2,18^. Healthy subjects are also recommended in bioequivalence studies to reduce pharmacokinetic and/or pharmacodynamic variability as they avoid potential confounding effects such as concomitant disease and medications^2,18^. Only male subjects were included as it was uncertain if the known insulin sensitivity in females during the menstrual cycle might affect the study results^2^. One concern with conducting insulin bioequivalence studies in healthy subjects is that the presence of endogenous insulin is not easily distinguished from exogenous insulin administration with available assays^2^, thereby interfering with the pharmacokinetic and dynamic measurements. In this respect, the bolus of the two rapid-acting insulin treatments should have adequately suppressed endogenous insulin for the duration of the clamp, meaning that no C-peptide correction was required^2^. In addition, endogenous insulin was suppressed by clamping blood glucose levels below the subject’s fasting glucose with plasma insulin aspart concentrations of both treatments measured using a validated and sensitive LC–MS/MS assay.

The insulin dose selected for this study under fasting conditions (0.3 U/kg) provided strong effects in the euglycemic clamp shown by a substantial increase in glucose demand reflected by the sizable GIR increase over the 10 h clamp duration and has been used in other similar rapid-acting insulin clamp studies^2,9,19^. The short duration of action and rapid clearance of insulin aspart meant that a clamp duration of 10 h was sufficient to adequately account for individual variations in insulin elimination and the duration of pharmacodynamic activity. It also minimized the time during which subjects were required to remain fasted and undergo the various clamp procedures^20^. The washout period of 7 to 18 days between the two dosing periods ensured that insulin concentrations were below the lower limit of quantification before the second treatment period.

The euglycemic clamp procedure in this study directly assessed the glucodynamic properties of SAR-Asp and NN-Asp-Jp. Clamp studies remain widely used to evaluate insulin action with the technique considered to be the reference standard for evaluating insulin sensitivity in human subjects^14,20^. They are also recommended for use in pharmacokinetic/dynamic studies that aim to show biosimilarity between two insulins^2^. The quality of the clamp performance is important for the interpretation of data. Here, the individual variability (CV%) of blood glucose per clamp was similar between treatments, with median CV values ranging from 5.9 to 7.1%. This was indicative of stable blood glucose concentrations during the clamp procedure with blood glucose levels during the clamp maintained close to their target level. These findings indicate that the GIR during the clamp was appropriate to measure the hypoglycemic effect of the two insulins.

Strengths of this study include the crossover design that allows for a within-subject comparison of the pharmacokinetic and pharmacodynamic response for the two insulins using the same subcutaneous doses, thereby enabling subjects to act as their own control. The glucose clamp technique and blinding of the investigator- and subject also avoided investigator-related bias. The requirement for standardized and well-controlled conditions (e.g., a long fasting period, administration of fixed insulin doses) is a recognized limitation of the euglycemic glucose clamp technique. However, the evaluation of the two treatments using a glucose clamp in phase 1 trials provides optimal sensitivity for detecting potential differences between the insulin treatments^9,14^. Establishing similar efficacy (i.e., change in HbA1c), safety and immunogenicity in real-life conditions is subsequently evaluated in larger phase 3 trials^14^.

Finally, rising insulin costs remain a concern for people with diabetes, their families and health care providers. The development of new biosimilar insulin products over the last few years, including rapid-acting insulins like reported here, has the potential to reduce drug treatment costs and thereby facilitate greater access of insulin treatment for people with diabetes. The reporting of such biosimilar trial data will increase the credibility of these valuable products and thereby provide benefit for physicians and their patients.

## Conclusion

This study showed that SAR-Asp had similar pharmacokinetic exposure and glucodynamic activity compared with Japanese-approved insulin aspart formulation (NN-Asp-Jp).

## Supplementary Information

Supplementary Information.

## Data Availability

The datasets generated during and/or analyzed during the current study are available from the corresponding author on reasonable request.
