# Supplementary material for: Pharmacokinetic and pharmacodynamic similarity between SAR341402 insulin aspart and Japan-approved NovoRapid in healthy Japanese subjects
Source: Sci Rep. 2021 Nov 25;11:22931. doi: 10.1038/s41598-021-02410-z (PMC8617249; doi:10.1038/s41598-021-02410-z)
Supplement: Supplementary file 1 — Supplementary Information. [file 41598_2021_2410_MOESM1_ESM.pdf]

Supplementary information

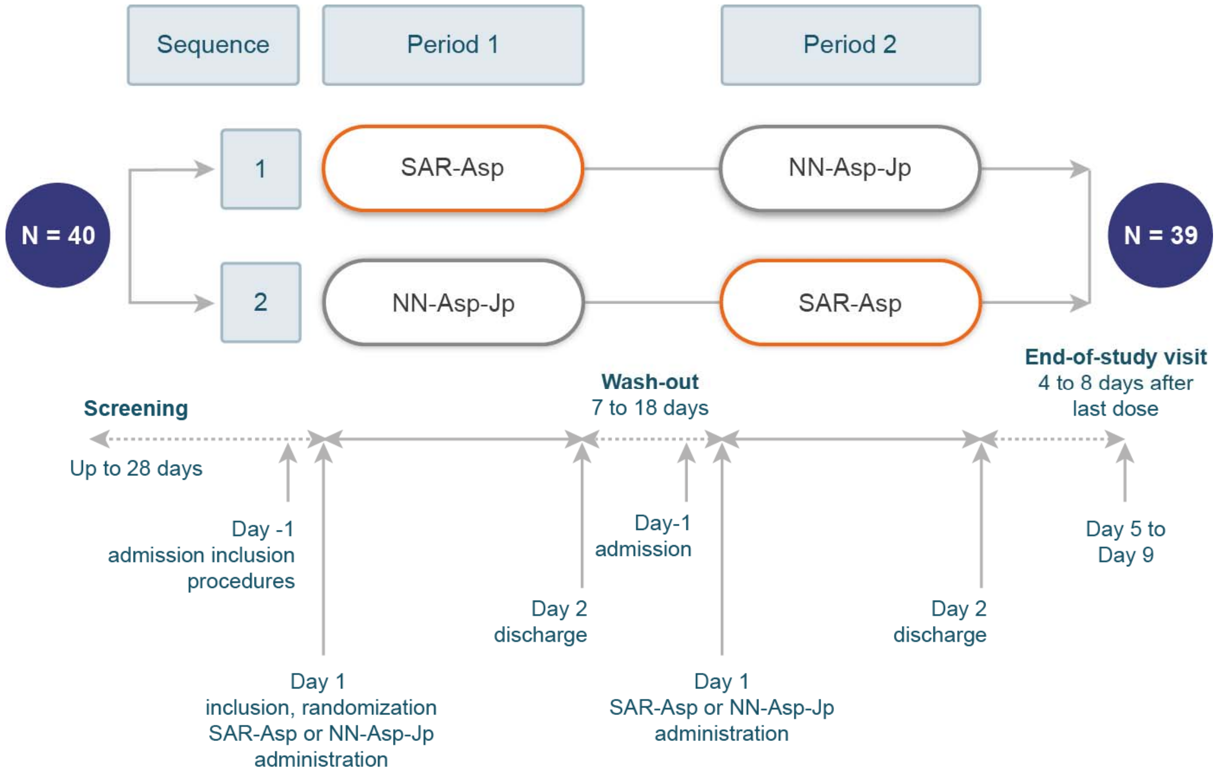

Supplementary Figure 1. Study design. Subjects were randomized to one of two treatment sequences as shown. In both periods, subjects received a single 0.3 U/kg dose of each treatment followed by a 10-h euglycemic clamp procedure. Subjects were discharged from the clinic one day following the clamp procedure.
